# ASSOCIATION OF SEDENTARY BEHAVIORS WITH PERCEIVED PHYSICAL FATIGABILITY

**DOI:** 10.1093/geroni/igae098.0236

**Published:** 2024-12-31

**Authors:** Reagan Garcia, Eileen Johnson, Peggy Cawthon, Barbara Nicklas, Bret Goodpaster, Anne Newman, Nancy Glynn

**Affiliations:** University of Pittsburgh School of Public Health, Pittsburgh, Pennsylvania, United States; Sutter Health, San Francisco, California, United States; California Pacific Medical Center, San Francisco, California, United States; Wake Forest University School of Medicine, Winston-Salem, North Carolina, United States; Advent Health, Orlando, Florida, United States; University of Pittsburgh, University of Pittsburgh, Pennsylvania, United States; University of Pittsburgh School of Public Health, Pittsburgh, Pennsylvania, United States

## Abstract

Greater perceived fatigability is a phenotype of poor energy regulation and is associated with physical activity, cardiovascular fitness, and mitochondrial energetics. However, it is unclear whether time spent in low energy expenditure activities, i.e. sedentary behaviors, contribute to greater perceptual feelings of fatigue. We examined whether sedentary behaviors were associated with perceived physical fatigability using cross-sectional data in the Study of Muscle, Mobility and Aging (SOMMA). Participants wore an activPAL on their right thigh for 7 consecutive 24-hour periods (valid wear=≥1 day with ≥10 hours). Total min/day sedentary time, time per sedentary bout (min/day), and number of sit-to-stand transitions were calculated using mean of out-of-bed intervals. Perceived fatigability was measured using the Pittsburgh Fatigability Scale Physical subscale (PFS, score 0-50, higher=greater fatigability, more severe=≥15). Logistic regressions were adjusted for age, sex, body mass index, Short Physical Performance Battery (range 0-12), and cost-capacity ratio (VO2 from 0.67 m/s 5-min walk/VO2peak *100). Participants (n=685; 76.4±5.1 years, 58% Women, 85.1% Non-Hispanic White, 54.9% PFS Physical score ≥15) had mean 619.1±113.1 min/day sedentary time, mean 15.2±5.8 min/day per sedentary bout, and 45.9±13.4 sit-to-stand transitions/day. One standard deviation higher mean sedentary time was associated with 26% greater odds of reporting more severe perceived physical fatigability after full adjustment (OR=1.26, 95% CI: 1.05-1.52). Mean time per sedentary bout and sit-to-stand transitions were not associated with perceived physical fatigability. Sedentary behavior has modest associations with perceived fatigability. Our work supports that interventions to reduce fatigability severity could be modestly enhanced if overall sedentary time is reduced.

